# A Rare Collision Tumor Composed of Follicular Lymphoma and Adenocarcinoma in the Ampulla of Vater: A Case Report

**DOI:** 10.1155/2014/530727

**Published:** 2014-04-28

**Authors:** Shioto Suzuki, Fumihiko Tanioka, Keisuke Inaba, Shingo Takatori, Hideto Ochiai, Shohachi Suzuki

**Affiliations:** ^1^Division of Pathology, Iwata City Hospital, 512-3 Ookubo, Iwata, Shizuoka 438-8550, Japan; ^2^Division of Gastroenterological Surgery, Iwata City Hospital, 512-3 Ookubo, Iwata, Shizuoka 438-8550, Japan; ^3^Division of Gastroenterology, Iwata City Hospital, 512-3 Ookubo, Iwata, Shizuoka 438-8550, Japan

## Abstract

The duodenum is infrequently affected by malignant lymphoma, and follicular lymphomas of the duodenum are rare histological subtypes. There are no reported cases of collision of follicular lymphoma and other tumors in the ampulla of Vater. A 57-year-old Japanese man presented with jaundice, and abdominal computed tomography revealed a tumor of the ampulla of Vater invading the pancreatic head with biliary dilatation and a thickened duodenal wall. The patient underwent subtotal stomach-preserving pancreaticoduodenectomy. Histopathology of the resected specimen revealed lymphoid follicular formations with small-to-medium-sized centrocyte-like cells and some centroblast-like cells. The atypical lymphoid cells were immunohistochemically positive for CD10, CD20, and CD79a but negative for CD5 and cyclin D1. BCL2 protein was highly expressed in the follicle centers. The diagnosis was duodenal follicular lymphoma, Grade 1. The follicular lymphoma, 40 mm in diameter, involved duodenal submucosa and regional lymph nodes without distant metastasis. This duodenal follicular lymphoma was partially overlapped by adenocarcinoma of the ampulla of Vater, measuring 25 × 20 mm, which involved the lower common bile duct, pancreas, and duodenum. We report the first case of a surgically treated collision tumor composed of a rare mass-forming follicular lymphoma and adenocarcinoma of the ampulla of Vater.

## 1. Introduction

Gastrointestinal (GI) primary lymphomas are the most frequent extranodal lymphomas, accounting for 30–40% [1]. The stomach is the most common site for lymphomas followed by the colorectal region and the terminal ileum [1]; the duodenum is less frequently affected [1]. Concerning histological subtypes, mucosa-associated lymphoid tissue (MALT) and high-grade B-cell lymphomas represent the vast majority, whereas follicular lymphomas (FLs) have a reported incidence of only 1.0–3.6% [2–4]. Primary GI-FLs usually arise in the small intestine, particularly in the terminal ileum [3], but duodenal FLs are rare. Three series of patients with duodenal FL [5–7] were recently reported as well as various previous case reports [1, 2, 8–11]. These reports characterized duodenal FLs as indolent and as having intermediate characteristics of MALT lymphomas and nodal follicular lymphomas [5–7]. Thus, a watch and wait approach appears to be the most sensible strategy [6]. Initially reported cases commonly occurred in women [2, 8, 9, 11], but this gender difference disappeared with a larger number of cases [5, 7]. Duodenal FLs frequently have multiple small nodular lesions, whereas mass lesions are very rare [4, 6]. Duodenal FLs, mainly small nodular lesions, were incidentally detected as synchronous tumors in 11 cases at gastroduodenoscopy during staging for preexisting tumors of other organs [6]. In addition, there is only one reported case of a rare synchronous tumor composed of FL, forming a mass lesion, of the ampulla of Vater and another tumor in another organ [8]. However, there is no reported case with collision, characterized by the coexistence of phenotypically and genotypically distinct tumors at the same site, of FL and other tumors in the ampulla of Vater.

We report the first unique case of a collision tumor, in which a rare mass-forming FL was incidentally diagnosed from a surgically treated specimen of cancer of the ampulla of Vater.

## 2. Case Presentation

**Figure 1:**
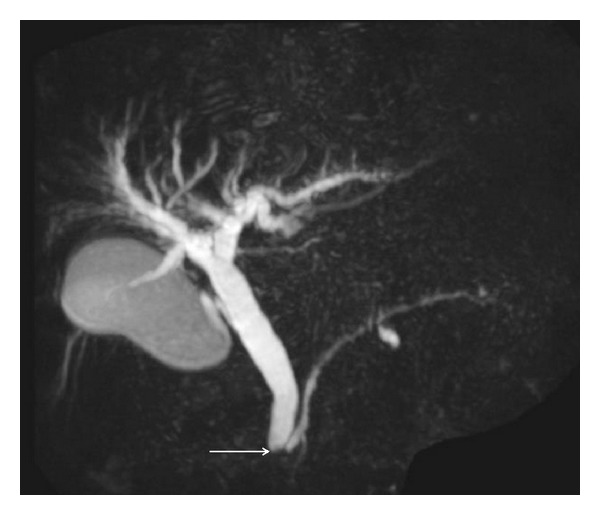
Magnetic resonance cholangiopancreatography showing dilated intrahepatic and extrahepatic bile ducts including the cystic duct and the gallbladder. The common bile duct measures 15 mm in diameter with abrupt concentric stenosis in its lower third and a cut-off point located 20 mm distally to the duodenal ampulla (arrow). The pancreatic duct is depicted as normal.

A 57-year-old Japanese man with no remarkable medical history was referred to our hospital with jaundice and a 1-week history of dark urine, as well as weight loss of 4 kg over 2 months. There was no abdominal pain or decreased appetite. On admission, the presence of jaundice and hepatic dysfunction were shown by aspartate aminotransferase (AST) 128 IU/L (normal, 8–40 IU/L), alanine aminotransferase (ALT) 232 IU/L (normal, 5–42 IU/L), alkaline phosphatase (ALP) 647 IU/L (normal, 117–335 IU/L), *γ*-glutamyl transpeptidase (*γ*GT) 1472 IU/L (normal, 16–73 IU/L), total bilirubin 15.3 mg/dL (normal, 0.3–1.3 mg/dL), and direct bilirubin 11.6 mg/dL (normal, <0.5 mg/dL). CA 19-9 was 101.7 U/mL (normal, <37 U/mL), while CEA was within the normal range. Contrast-enhanced abdominal computed tomography (CT) revealed (i) dilatation of the biliary tree, (ii) tumor of the papilla of Vater invading the pancreatic head, measuring 18 mm in diameter, and (iii) a thickened duodenal wall. Neither lymph node enlargement nor splenomegaly was demonstrated. Contrast-enhanced magnetic resonance imaging (MRI) revealed an irregular tumor corresponding to the ampulla of Vater with infiltration to the adjacent duodenal wall, involvement of the lower bile duct, and dilated intrahepatic and extrahepatic bile ducts, including the cystic duct and the gallbladder. The common bile duct (CBD) measured 15 mm in diameter with an abrupt concentric stenosis in its lower third and a cut-off point located 20 mm distally to the duodenal ampulla (Figure 1). The pancreatic duct was depicted as normal.

**Figure 2:**
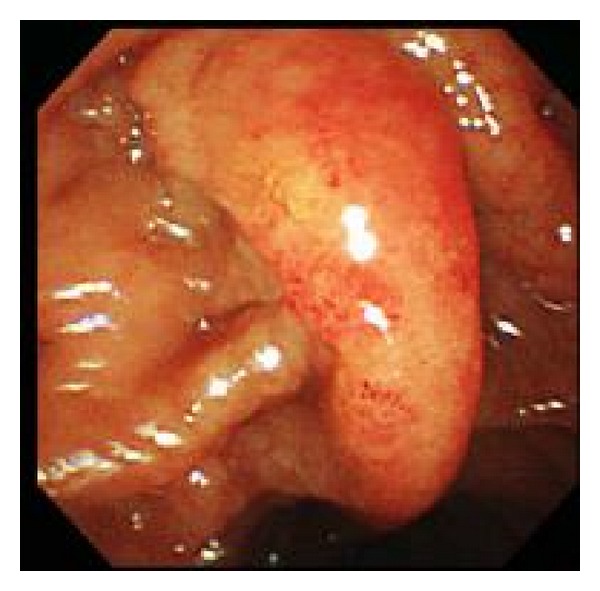
Endoscopic appearance showing the duodenal papilla covered with whitish tiny granules. The tumor of the ampulla of Vater is not exposed.

The patient underwent endoscopic retrograde cholangiopancreatography (ERCP), and a nasobiliary drainage tube was placed. The duodenal papilla was depicted as being covered with whitish tiny granules, whereas the tumor of the ampulla of Vater was not exposed (Figure 2). Biopsy samples taken from duodenal mucosa around the duodenal papilla revealed only lymphocyte infiltration without a nodular pattern and no definite diagnosis was made. However, adenocarcinoma was cytologically suggested in the collected bile juice. Taking these findings into consideration, subtotal stomach-preserving pancreaticoduodenectomy (SSPPD) was performed with regional lymph node resection for the tumor of the ampulla of Vater.

## 3. Histology

**Figure 3:**
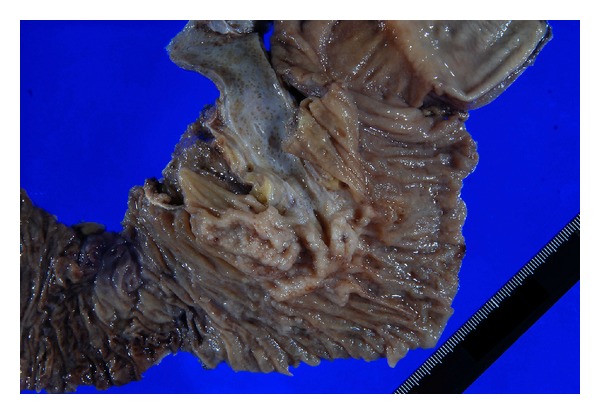
Macroscopic findings of the tumor. The tumor of the ampulla of Vater forms a mass with ulceration and multiple small nodular lesions are found around it.

**Figure 4:**
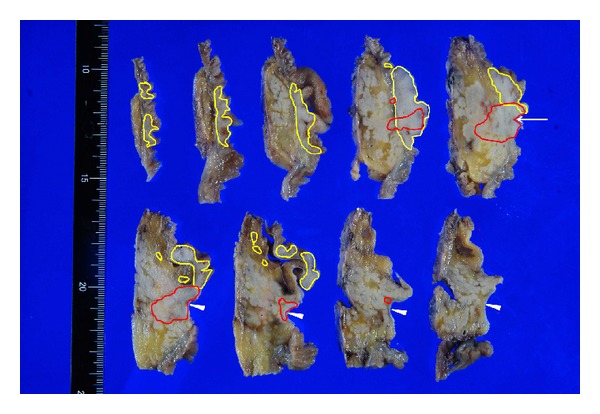
Cross-sections of the resected specimen. The tumor is composed of follicular lymphoma (enclosed by curved yellow line) and adenocarcinoma (enclosed by curved red line) of the ampulla of Vater (arrow). Adenocarcinoma includes the lower part of the common bile duct (arrowheads).

The tumor of the ampulla of Vater was macroscopically observed as an ulcerative mass accompanied by surrounding multiple small nodular lesions (Figures 3 and 4).

**Figure 5:**
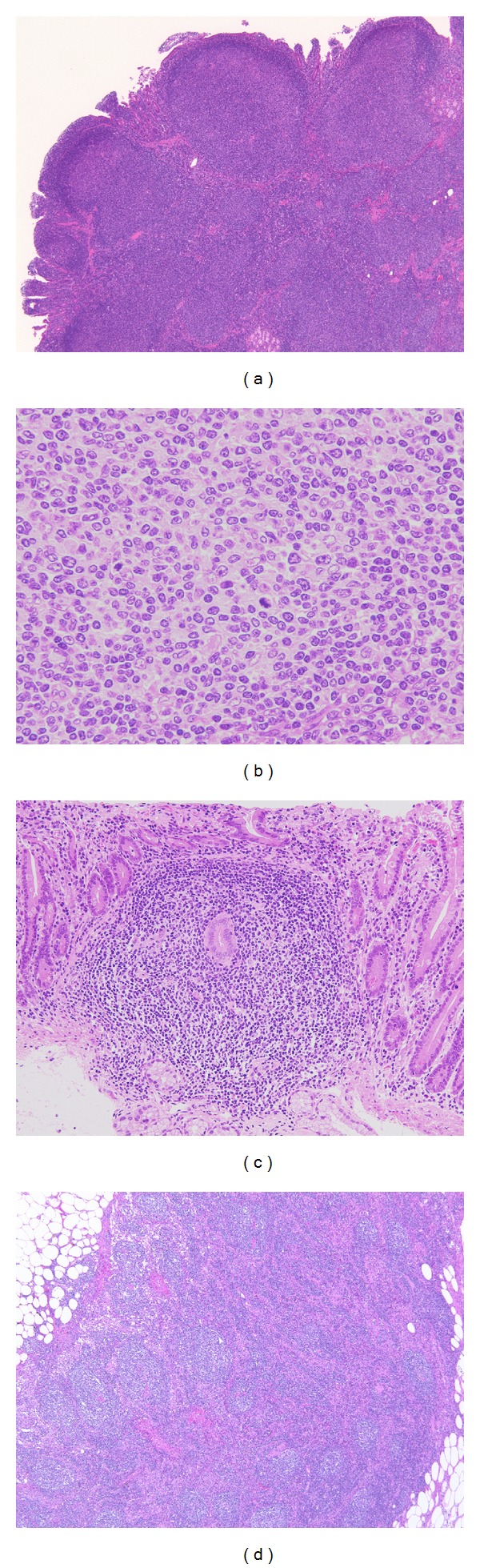
Representative histologic images of the follicular lymphoma. ((a)–(d)) The lesion reveals lymphoid follicular formations (a) with small-to-medium-sized centrocyte-like cells and a few centroblast-like cells (b). Small nodular lesions around the main tumor also consist of follicular lymphoma (c). Regional lymph nodes are partially involved by follicular lymphoma (d).

**Figure 6:**
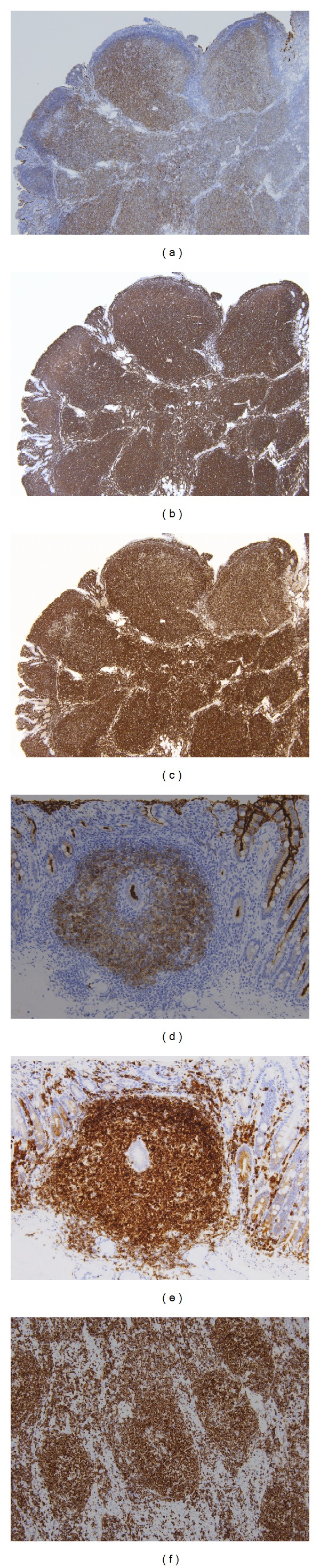
Immunohistochemical findings of follicular lymphoma. ((a)–(f)) Atypical lymphoid cells are positive for CD10 (a) and CD20 (b). BCL2 protein is intensively expressed in the centers of the follicles (c). Atypical lymphocytes in small nodular lesions (Figure 5(c)) around the mass of the ampulla of Vater are immunohistochemically positive for CD10 (d) and BCL2 (e). In regional lymph nodes (Figure 5(d)) in the duodenum, BCL2 protein is intensively expressed in the centers of the follicles (f).

**Figure 7:**
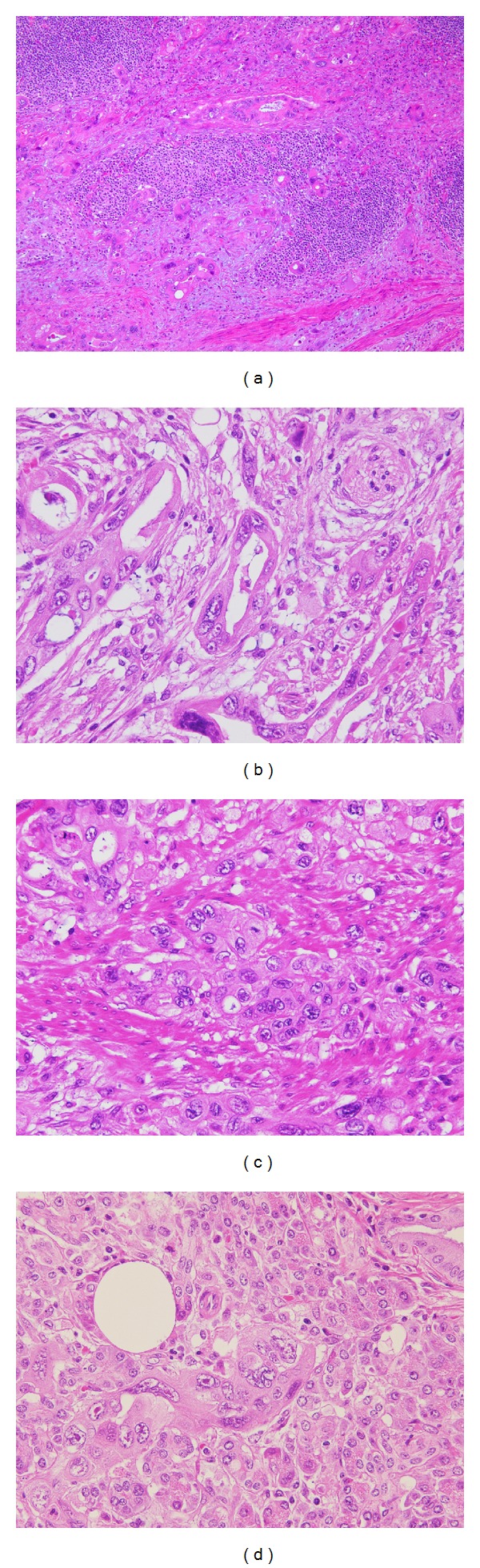
Representative histologic images of adenocarcinoma of the ampulla of Vater. ((a)–(d)) Follicles of follicular lymphoma are partially invaded by adenocarcinoma (a). Adenocarcinoma shows various patterns, including tubular (b), cribriform, and trabecular structure and nests (c), which invade the pancreas (d).

Histopathological study of the tumor unexpectedly revealed that the lesion was composed of two independent neoplasms. The surface part of the tumor showed lymphoid follicular formations (Figure 5(a)) with small-to-medium-sized centrocyte-like cells and a few centroblast-like cells (Figure 5(b)). Follicles lacked polarity. Immunohistochemical study demonstrated that the atypical lymphoid cells were positive for CD10 (Figure 6(a)), CD20 (Figure 6(b)), and CD79a but negative for CD5 and cyclin D1. BCL2 protein was intensively expressed in the centers of the follicles (Figure 6(c)). Based on these findings, the diagnosis of follicular lymphoma, Grade 1, was made [2, 4], according to the World Health Organization (WHO) classification. The duodenal FL formed a mass, measuring 40 mm in diameter, which involved the duodenal mucosa and submucosa (enclosed by curved yellow line in Figure 4). Atypical lymphocytes in small nodular lesions (Figure 5(c)) around this mass and regional lymph nodes (Figure 5(d)) in the duodenum were also immunohistochemically positive for CD10 (Figure 6(d)), CD20, CD79a, and BCL2 (Figures 6(e) and 6(f)). These results showed that the small nodular lesions also consisted of FL, and regional lymph nodes were partially involved. No distant metastasis was found. The mass, consisting of FL, was partially invaded by adenocarcinoma (Figure 7(a)). Adenocarcinoma showed various patterns, including tubular (Figure 7(b)), cribriform, and trabecular structures and nests (Figure 7(c)), classified as the pancreatobiliary type. Adenocarcinoma of the ampulla of Vater formed a solid tumor (enclosed by curved red line in Figure 4), 25 × 20 mm, involving the lower CBD for 20 mm in length, pancreas (Figure 7(d)), and duodenum, with metastasis to regional lymph node (1/28 in total). The adenocarcinoma of the ampulla of Vater was classified as stage IIB (pT3, pN1, and M0).

The patient was discharged one month after the operation without critical complications. S-1 oral intake was selected as adjuvant chemotherapy targeting the cancer of the ampulla of Vater. Nine months after the operation, multiple liver metastases were detected. CT scans suggested that those lesions were derived from adenocarcinoma of the ampulla of Vater rather than duodenal FL. The patient received systemic chemotherapy including gemcitabine; however, multiple lung metastases with pleural effusion and peritoneal dissemination appeared. Metastatic adenocarcinoma was cytologically suggested in the pleural effusion, whereas involvement of FL was not suggested. One year after the operation, the patient died of multiple organ failure with primary disease progression. Autopsy was not permitted.

## 4. Discussion

Previous studies characterized primary duodenal FL as a markedly indolent FL variant, which, even when left untreated, very rarely disseminates and does not transform to high-grade disease [2, 6, 7, 12]. In our case, lymphoma cell infiltration was rather superficial and was confined to the submucosal layer, whereas the regional lymph nodes were involved partially without distant metastasis. These findings were also observed in reported cases [2, 4]. Although our patient died of multiple organ failure with multiple liver and pulmonary metastases, CT scans suggested that those lesions were derived from adenocarcinoma of the ampulla of Vater rather than duodenal FL. Furthermore, metastatic adenocarcinoma was cytologically detected in the pleural effusion, whereas involvement of FL was not suggested. Thus, FL might not have been the direct cause of death, although the resected specimen showed that FL is comprised of a larger part of the primary lesion than adenocarcinoma.

Diagnosing FL of the duodenum is not easy [2, 5]. In a previous study [2], three of five patients with FL were diagnosed from the surgically resected material but not the biopsy specimens. In our case, a biopsy specimen, taken from the duodenum, showed only scattered lymphocyte infiltration without a follicular pattern, so it did not seem to be taken from the lesion of follicular lymphoma. Thus, no definitive diagnosis was made for duodenal FL prior to surgery, as in previous reports [2]. In our surgical specimen, atypical lymphoid cells were immunohistochemically positive for CD10, CD20, and CD79a but negative for CD5 and cyclin D1. BCL2 protein was intensively expressed in the centers of the follicles. These results were compatible with those in FLs, in which both CD10 and BCL2 are expressed [4]. In contrast, MALT lymphoma is negative for CD10 and only occasionally positive for BCL2 [4]. In addition, mantle cell lymphoma expresses cyclin D1 and BCL2 but negative for CD10 [2, 4]. Immunohistochemical findings were useful for distinguishing our case from MALT lymphoma and mantle cell lymphoma [2, 4].

Careful clinical examination, including the capsule of double-balloon enteroscopy, seems to increase the number of cases diagnosed as duodenal FLs [5–7]. Endoscopically, multiple small polypoid lesions, usually measuring 1 to 5 mm, in the descending part of the duodenum are the most frequent findings, with clustering around the ampulla of Vater in patients with duodenal FL [4, 6]. On the other hand, a solitary tumor, as in our case, was only observed in 4–15% of patients [6]. In our case, FL formed a mass, measuring 40 mm in diameter, which was relatively large compared with those reported, with the largest measuring 40 mm in diameter [4, 6]. Since duodenal FL tends to grow slowly [4], these findings suggest its existence prior to adenocarcinoma of the ampulla of Vater, which grows rapidly. Biliary obstruction secondary to lymphoma is rare, with an incidence of only 0.6% of 1123 patients with malignant biliary obstruction [13]. In our case, adenocarcinoma directly invaded the lower CBD for 20 mm in length, whereas FL was located mainly in the duodenal mucosa and submucosal layer and involved the papilla of Vater overlapping the adenocarcinoma. These findings implied that biliary obstruction might have been caused mainly by the bile duct involvement of adenocarcinoma, which grew more rapidly than the preexisting FL, which grew slowly without symptoms.

The presenting symptoms leading to gastroduodenoscopy were not related to the duodenal FL of reported cases [4, 6, 10]. Most primary duodenal FLs were found incidentally at routine medical checkups [4, 7, 10] or found as part of a synchronous tumor at staging for other neoplastic conditions [6]. Synchronously diagnosed cancers may be the result of a “lead time bias” since the second tumor may be detected while asymptomatic because of increased medical scrutiny [14]. There is only one reported case of a rare synchronous double tumor in the biliary tract, which was composed of FL of the ampulla of Vater and an endocrine tumor of the common bile duct [8], although this was not a collision tumor. On the other hand, although a relatively high incidence of other malignancies in patients with carcinoma of the ampulla of Vater was revealed, FL was not included in the associated malignancies [15, 16].

There is no reported case of a collision tumor in the ampulla of Vater, composed of FL and other tumors, and its tumorigenesis is uncertain. Niu et al. [15] hypothesized that several possible factors may lead to the tumorigenesis of collision cancer: (i) a carcinogenic agent can affect multiple targets simultaneously; (ii) decrease of the systemic and local immunodefense system after the development of one tumor facilitates the occurrence of another tumor; and (iii) dysfunction of the tumor suppressor mutated gene leads to inadequate repair of a gene mutation, resulting in the formation of multiple tumors.

Concerning carcinogenesis, a high incidence of biliary tract cancer has been revealed, particularly in pancreaticobiliary maljunction [17]. A mixture of pancreatic juice and bile is constantly being produced, and when bacterial infections and an increase in pressure in either the pancreatic duct or the bile duct are also present, pancreatic enzymes easily become activated, producing a variety of carcinogenic agents. Because of this, the biliary mucosa is repeatedly damaged and repaired, which causes the acceleration of cell proliferative activity and multiple gene mutations [17]. In addition, recent studies [2, 5] reported that the incidence of FL was high in the duodenum compared with other portions of the GI tract. Yoshino et al. [2] speculated that direct stimulation of bile and pancreatic juice in the duodenal mucosa might result in its duodenal predilection.

Collectively, these hypotheses could support the cause of the collision tumor in our case. For example, direct stimulation of bile and pancreatic juice in the duodenal mucosa might affect both lymphocytes and the epithelium of the ampulla of Vater. Development of FL, for example, forming a relatively large mass lesion as a rare form as in our case, might decrease the local immunodefense system and facilitate the tumorigenesis of adenocarcinoma from the affected epithelium of the ampulla of Vater. In this line, the tumorigenesis of only one reported case [8] of a synchronous tumor, composed of a carcinoid tumor of CBD and FL of the ampulla of Vater, might be partially explained. Direct stimulation of bile and pancreatic juice in the duodenal mucosa or the decrease of the local immunodefense system associated with the development of a carcinoid tumor might facilitate the occurrence of FL, which formed a rare mass lesion. Further tumorigenetic studies, including the accumulation of case reports, are necessary to clarify the etiology of the frequent development of other malignancies, for example, follicular lymphoma as in our case, in patients with carcinoma of the ampulla of Vater.

## 5. Conclusion

We herein present the first unique case of symptoms due to cancer of the ampulla of Vater, whereas FL, an asymptomatic and infrequent tumor, was incidentally diagnosed as part of a collision tumor in a resected specimen of the cancer.
